# Biased Perceptions and Personality Traits Attribution: Cognitive Aspects in Future Interventions for Organizations

**DOI:** 10.3389/fpsyg.2018.02472

**Published:** 2019-01-15

**Authors:** Silvia Riva, Ezekiel Chinyio, Paul Hampton

**Affiliations:** Faculty of Science and Engineering, University of Wolverhampton, Wolverhampton, United Kingdom

**Keywords:** gender, implicit personality theory, bias, social cognitive theory, nudging, differentiation

## Abstract

In most European countries, the proportion of females and males pursuing a career in Technology and Engineering is quite different. The under-representation of women in these jobs may be attributable to a variety of factors, one of which could be the negative and stereotyped perception of these work sectors as unsuitable for women. The purpose of this study was to determine whether stereotyped perceptions impact the job representation of males and females in the Construction Industry, which is a particularly male-dominated work sector. Three construction organizations in the West Midlands (United Kingdom) were studied by means of ethnographic interviews and observations. Three (6.7%) of the 45 research participants (mean age 44.3) were women (focusing only in people working in Construction sites). There was a high differentiation of activities between the males and females. Biased perceptions and personality traits attribution played a fundamental role in such a differentiation. Despite some main limitations (low generalisability, lack of longitudinal findings), this study focused on some important practical implications for current work policies: changing the mindsets of people (starting from school age), using new flexible strategies and creating the role of internal advocates. The findings provide definitive evidence of the need to increase the promotion of social communication and public campaigns on gender equalities in male-dominated work sectors, taking into account the cognitive processes behind gender differences. The findings also give new hints on re-thinking the contribution of Psychology, particularly Cognitive Psychology, in fields with allegedly wide gender gaps.

## Introduction

The percentage of women pursuing a career in Technology and Engineering is still very low (Wang and Degol, 2017; Stoet and Geary, 2018) at approximately 8–10% in the EU (Clarke et al., 2015). The Construction Industry (CI) employs approximately 2.1 million people in the United Kingdom, and its employment gender gap is one of the highest (Rhodes, 2015). While few studies have explored this gender gap in the CI (e.g., Gannon et al., 2007; Ericksen and Schultheiss, 2009; Arena et al., 2015) there is scope for much more.

There are stereotyped perceptions against women in the CI, attributable to their suitability for such careers (Jussim, 2012; Olmos-Peñuela et al., 2014). The sparse and vague data about women’s participation in construction (Gov.UK, 2015) are not framed in any particular theoretical background. Meanwhile, cognitive Psychology represents an interesting frame of reference for understanding the phenomenon of stereotyped perceptions: how people often form impressions of others using gender and evaluate actions and performance on this basis (Laws, 1977; Hyde and Linn, 1988). Stereotypical influences are generated by different socialization agents (in primis, the family), and start in early childhood. The two paradigms of Implicit Personality Theory and the Social Cognitive theory are very useful for understanding these influences.

The *implicit personality theory* describes the specific patterns and biases a person adopts when developing impressions based on a limited amount of initial information about an unfamiliar person (Schneider, 1973). Parts of the impression formation process are context-dependent, while others cut across a variety of situations. In this perspective, gender conceptions and role behaviors can be based on a structured set of inferential relations that link personal attributes to the social categories of gender (Ashmore and Del Boca, 1979; Hamilton, 2015; Kray et al., 2017).

The *social cognitive theory* (Bandura, 1986) has its roots in cognitive psychology and biology, and emphasizes how the individual is a “maker of meanings” (Schunk, 1989; Bussey and Bandura, 1999; Lent et al., 2002; Leaper, 2015). In this theory, gender development is promoted by three modes:

(I) *modeling*, where the great majority of information about gender is transmitted by models of an individual’s direct environment, such as family, peers and other important people in their social and occupational life. Also, the media and internet persistently model gender-affiliated roles and behaviors.
(II) *enactive experience*, which is the process of internalizing information about what is proper behavior for certain genders by the reactions it produces in others.
(III) *direct tuition*, where people attempt to influence gender behaviors in others by using either approval and consent or disapproval and displeasure.

This paper reports on stereotyped gender perceptions in the CI. It is based on a larger EU study on stress in the CI (INSTINCT; Project ID: 703236, H2020-EU.1.3.2. – Nurturing excellence by means of cross-border and cross-sector mobility).

## Methods

The research was conducted using ethnography. Ethical approval was obtained from our employer (University) and informed consent and privacy statements were obtained from all the participants. An intensive effort was made to grasp the participants’ perspectives in the broad fieldwork which continued until saturation (Smith, 2001) was adjudged.

Four approaches were utilized to collect data: observation, field notes, two-prong interviews (unstructured and semi-structured) and colloquial discussion. These different methods facilitated an in-depth acquisition of knowledge through the intersection or convergence principle (Denzin, 1997), and triangulation of data. Questions asked concerned gender issues, job activities, problems experienced, stress, and mental issues in the workplace. Using a “grounded” approach, the researchers began with prefigured problems (Hammersley and Atkinson, 2007) and scholarly curiosity (Smith, 2001) but specific questions were not selected prior to immersion, in order to ensure a better validity of the findings.

### Ethnographic Data Acquisition and Analysis

Several visits to work places and meetings were held with 45 people, and 55 observations were limited to people working on open sites (Table 1). During the ethnographic period, which lasted 6 months, we met only 4 women: three of these (3/45, 7%) were interviewed and all four (4/55, 7%) were observed while at work. The observations made, interview transcriptions and field notes were content analyzed following rules and procedures (Denzin, 1997) and persistently discussed between all the authors.

**Table 1:** Methodological considerations.

Sampling	Involvement	Descriptors used (Reeves et al., 2008, p. 512)	Data analysis
*Approach:* Non- probability sample *Method:* Convenience *People excluded*:^∗^ *Criterion*: Saturation	*People:* 45 *Interviews:* 18 *Observational frame:* 55 *Places:* 12 places in 3 industries *Times:* 2–4 h/ observation in 6 months (Nov–May 2018) *Context:* 10 sites and 2 offices	*Space*: Physical layout of the place(s) *Actor*: Range of people involved *Activity*: A set of related activities that occur *Object*: The physical things that are present *Act*: Single actions people undertake *Event*: Activities that people carry out *Time*: The sequencing of events that occur *Goal*: Things that people are trying to accomplish *Feeling*: Emotions felt and expressed	*Roadmap used:* • Coding for descriptive labels • Sorting for pattern • Identifying outliers • Generalizing constructs and theories


*^∗^2 = refused participation; 5 = consultants, external to organizations.*

## Results

The activities of males and females were found to be strongly differentiated, as shown in Table 2: women were lopsidedly tasked with only negotiation and consultancy activities.

**Table 2:** Activities observed in the study.

Activity observed	Performed by (Gender):	
	Males	Females
Contracts	✓	
Review of document	✓	✓
Contacts with occupiers/tenants	✓	✓
Bids and contracts	✓	
Grading and building permits	✓	
Site work	✓	
Foundation	✓	
Rough carpentry	✓	
Concrete slabs	✓	
Heating, ventilation and air conditioning	✓	
Plumbing work	✓	
Electrical work	✓	
Roofing	✓	
Exterior finishes	✓	
Insulation	✓	
Drywalling	✓	
Floor finishes	✓	
Painting	✓	✓
Final walk-through	✓	


A typical description of a construction worker is: a man with physical skills, such as strength and endurance, and the ability to cope with different environmental difficulties (e.g., weather conditions, risky environments) and manage power relationships (leadership, taking hazardous decisions). Males occupied all of the managerial positions observed. It was often reported that women had managerial positions at offices and not on sites. However, these office positions were often operating under higher positions occupied by men. This differentiation was often attributed to gender traits and personality: the idea that men are more goal-oriented, charismatic and directive, while women are more sensitive, cooperative and responsive. In line with the implicit personality theory (Ashmore and Del Boca, 1979; Hamilton, 2015; Kray et al., 2017), these descriptions reflect the gender stereotyping of allocating *softer* activities (e.g., communication, negotiation and consultancy) to women and *harder* activities (including physical jobs, leadership roles) to men.

The study also observed differentiation in the impact of work activities on the level of stress perceived at work. While stress and concerns in women were more related to their ability to communicate and negotiate (relational capabilities), stress in men was much more related to time management, changes to plans and adhering to strict deadlines (organizational) and manual capabilities.

Consolidated experiences and tradition also informed role differentiation. As reported by a worker, “*Building was in the males of our family…. My grandfather was a bricklayer, my father was a bricklayer, so it was in the blood.*” Following the social cognitive perspective, the gender-based relations in construction work were developed in both routine, day-to-day interactions and legitimized within the larger social and organizational context in which divisions of labor, power and culture were defined. Indeed, the study found a paradigm of gender differences built by modeling (past models of male and female activities in construction influenced the future roles), enactive experience (female workers were always recruited for certain activities and not others, in a repetitive and recursive model), and direct tuition (cultural organization and practices always proposed a clear distinction between women’s and men’s tasks).

## Discussion

Our study found a marked diversity in the roles and activities of men and women, suggesting the presence stereotyping, in line with the implicit theory of personality (Ashmore and Del Boca, 1979; Hamilton, 2015; Kray et al., 2017). The data also reflected a fallacious cultural representation of gender, in line with the social cognitive perspective (Schunk, 1989; Bussey and Bandura, 1999; Lent et al., 2002; Leaper, 2015). The results align with current literature (e.g., Ramaci et al., 2017; Santisi et al., 2018).

There are relatively few Psychology studies on the construction sector. Hence, one novelty of this study is the appraisal of construction workers through the lens of psychology. Another novelty is the differentiation of the perceived level of stress and coping mechanisms: women had to cope more with their communication skills while men had to cope more with their organizational skills.

Although this study does not indicate a definitive solution for overcoming gender inequalities, it highlights the importance of considering reasoning processes, associated bias and fallacious inferences when promoting policies of gender equality in the workplace. In some countries, such as the United Kingdom, women have been positively encouraged through, for instance, proactive recruitment campaigns and communication activities using positive images of tradeswomen and active enforcement of equal opportunity policies (CSC, 2018; RISE, 2018). However, these campaigns have had little impact. Hence additional two things are needed: (1) a strategic goal of increasing the number of women in construction workplaces; and (2) an immediate plan for reducing bias and stereotyping in the construction sector. Cognitive psychological research suggests that many biases disappear when people work and cooperate with each other (e.g., Renzi et al., 2016). Promoting actions in this direction may help to reduce the gender gap in the CI.

Job rotation (Lu and Yang, 2015) may also facilitate a more gender-balanced CI environment, as it can permit different job assignments for broader work experience, leading to future managerial positions both for males and females (Sato et al., 2017). Another worthwhile concept is “job crafting,” which promotes more equal opportunities for men and women and specific proactive behaviors in which employees initiate changes in the levels of job demands and resources (Tims and Bakker, 2010). Job crafting provides a proactive coping mechanism for reducing stress and burnout, and appears to be new in the CI (Singh and Singh, 2018).

In Behavioral Science and Cognitive Psychology, “nudging” is “any factor that significantly alters the behavior of humans” (Thaler and Sunstein, 2003, p. 175) through indirect suggestions that can influence motives, incentives and change-promotion. Nudges can change organizational practices and the way people are hired, promote employees in different ways and create a more equal playing field for men and women. For example, the human resources departments of CI organizations could set a nudging “default option” (Thaler and Sunstein, 2003) of prioritizing the recruitment of women whenever the percentage of their male employees disproportionately exceeds a certain threshold.

Last but not least, it is important to promote more thoughtful policies on the work-family balance. The preponderance of men in the CI is explained by the lack of trained women, and sometimes the lack of supporting policies for women at work, which, for instance, makes it difficult for women to re-enter the labor market after a career break due to childbearing or other family reasons (Lingard and Lin, 2004; Korpi et al., 2013; Budig et al., 2016).

## Conclusion

This is an initial study and the current findings require further investigation. In fact, it must be acknowledged that the context explored might not be representative of the whole CI. In addition, it is important to compare our results with longitudinal studies in order to evaluate how career perspectives develop over time. In spite of these limitations, this study has some practical implications for future work policies that promote gender equality. In particular, the study is intended to highlight three points:

• Changing mind-sets starting from school age: gender diversity and equality have to be promoted better at school and during early education.
• Industries need to create more flexible strategies for women including working from home, job-sharing and consulting assignments.
• Promoting gender equality by creating internal advocates. Having employees who champion this advocacy means they can oversee short-term actions and sustain long-term ones toward equality.

This is one of the first studies to be conducted in the CI with a psychological perspective. The study identified stereotypical perceptions and behaviors in the construction sector. Stress affects many construction workers and the way men respond to it is different from the way women do. The results and suggestions from this study should be factored in work-family policy reflections. Male-dominated work sectors, such as the CI, have to think more creatively about the promotion of gender participation in their environments, and Psychology can give new insights and perspectives to rethink equality and gender-balanced contributions.

## Author Contributions

SR carried out the ethnography. SR and EC performed the analysis and drafted the manuscript. PH participated in the design of the study and drafted the manuscript. All authors read and approved the final manuscript.

## Conflict of Interest Statement

The authors declare that the research was conducted in the absence of any commercial or financial relationships that could be construed as a potential conflict of interest.
